# Bilateral Hutch diverticula in an elderly male: Revelation of an unknown past

**DOI:** 10.4102/sajr.v24i1.1963

**Published:** 2020-12-11

**Authors:** Siddhi Chawla, Shuchi Bhatt, Anupama Tandon, Gaurav Meena, Saumya Dangwal

**Affiliations:** 1 Department of Radiology, Faculty Medical Sciences, University College of Medical Sciences and GTB Hospital, University of Delhi, Delhi, India

**Keywords:** bladder diverticulum, Hutch diverticulum, ultrasound, intravenous pyelography, computed tomography

## Abstract

Hutch diverticulum is a congenital diverticulum of the urinary bladder, reported infrequently in children and rare amongst adults. We present a 60-year-old male patient with bilateral Hutch diverticula, detected incidentally during an abdominal ultrasound examination performed for blunt abdominal trauma. This rare case highlights an unusual incidental presentation and opportunity to learn how to differentiate it from acquired bladder diverticula. The available treatment options are also discussed varying from simple follow-up to aggressive surgery.

## Introduction

Urinary bladder (UB) diverticula are herniations of the bladder mucosa through muscular fibers of the bladder wall^1^ resulting in a thin-walled structure that poorly empties during micturition. They are classified as congenital or acquired. Congenital diverticula are reported infrequently in children and are ever rarer in adults^2^ Their incidence is reported to be 1.7%^3^ and peaks in children below 10 years of age.^4^ Ninety percent of congenital bladder diverticula are located superolateral to the ureteral orifice, in proximity to the uretero-vesical junction.^5^ Unlike the acquired adult form, in which outlet obstruction or neurogenic dysfunction is almost always present, congenital bladder diverticula result from hypoplasia of the muscular layer of the bladder wall.^1^ It may be unilateral and infrequently bilateral. Patients may remain asymptomatic or may present with symptoms of urinary retention, obstruction, voiding dysfunction, recurrent urinary tract infection and calculi formation in the diverticulum.^6^ The symptoms are more pronounced in patients with a large diverticulum, whereas cases with a small diverticulum may remain undiagnosed. We present the rare case of bilateral asymptomatic congenital bladder diverticula discovered incidentally in an elderly male patient.

## Case presentation

**FIGURE 1:**
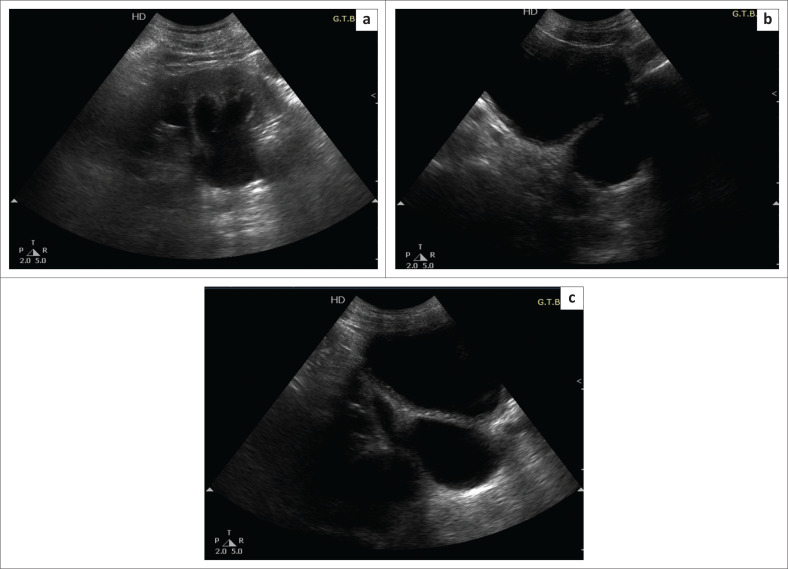
Ultrasound images show (a) gross hydronephrosis affecting the left kidney. (b) A well-defined outpouching in region of left vesicoureteric junction. (c) The left ureter is seen to open into the outpouching. Distal ureter appears dilated.

**FIGURE 2:**
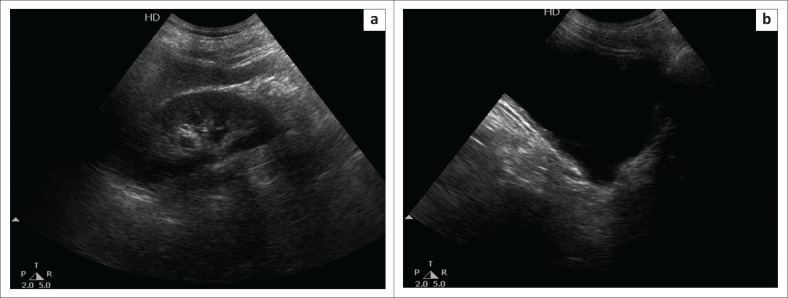
(a) Ultrasound images of the normal right kidney (b) a small diverticulum is present in the region of the right vesicoureteric junction. A normal calibre right ureter is seen opening into it.

A 60-year-old male patient presented with a history of a fall, followed by acute pain in the abdomen for which he was attended to in the emergency department. The patient was sent for Focused Assessment with Sonography for Trauma (USG FAST), which was negative. Incidental note was made of left hydronephrosis (Figure 1) with a large well-defined outpouching from the left postero-lateral wall of the UB. It measured 4 cm × 5 cm. A similar smaller (1.2 cm × 1.6 cm) outpouching was also seen on the right side of the UB (Figure 2). The bilateral vesicoureteric junctions (VUJs) could not be seen separately. The wall of the bladder was mildly thickened but there was no evidence of trabeculations. The prostate gland measured 24 cubic centimetre (cc) with a normal shape and echotexture. There was only 40 cc of insignificant post-void residual urine in both the UB and the left diverticula together.

Focused detailed history taking revealed a complaint of difficulty in micturition with a subsequent sensation of incomplete voiding over the previous 1–2 years, resulting in a repetitive need to pass urine. Intermittent dull pain was also present in left flank. There was no history of passage of blood or pus in the urine, urgency for micturition, hesitancy, poor stream or terminal dribbling of urine. There was no past history of any long standing illness in the patient.

**FIGURE 3:**
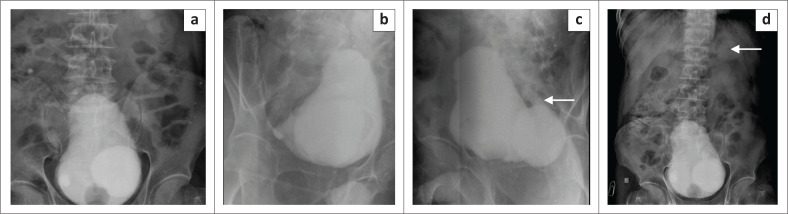
Sequential images of a micturating cysto-urethrogram: (a) Anteroposterior image demonstrates well-defined outpouchings bilaterally in the postero-lateral wall of the bladder. (b) Right anterior oblique shows the right contrast-filled outpouching. (c, d) Left anterior oblique and supine images reveal the left diverticulum and reflux of contrast into the left dilated, tortuous ureter, extending up to the pelvicalyceal system, which appears hydronephrotic, consistent with (Grade 5) vesicoureteric reflux.

As the patient’s complains were directed towards a lower urinary tract infection, retrograde urethrography (RGU) and micturating cysto-urethrogram (MCU) were performed. The RGU was unremarkable. The MCU (Figure 3) demonstrated a well-defined outpouching from the postero-lateral wall of the UB on both sides, the left being significantly larger in size and more well-defined than the right. There was grade 5 vesicoureteric reflux (VUR) on the left side causing dilatation and tortuousity of the entire left ureter and moderate left hydronephrosis. No reflux was seen on the right.

**FIGURE 4:**
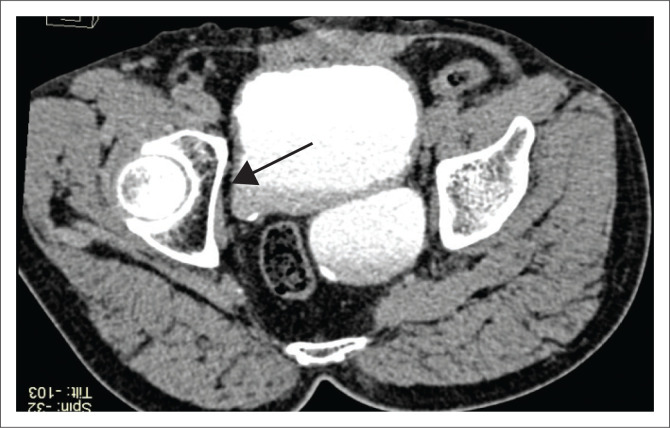
Axial multiplanar reformatted section shows the diverticula bilaterally at the vesicoureteric junctions. The left contrast-filled diverticulum is larger and is causing mild compression and displacement of the rectum. The right is small and is not filled with contrast (arrow).

**FIGURE 5:**
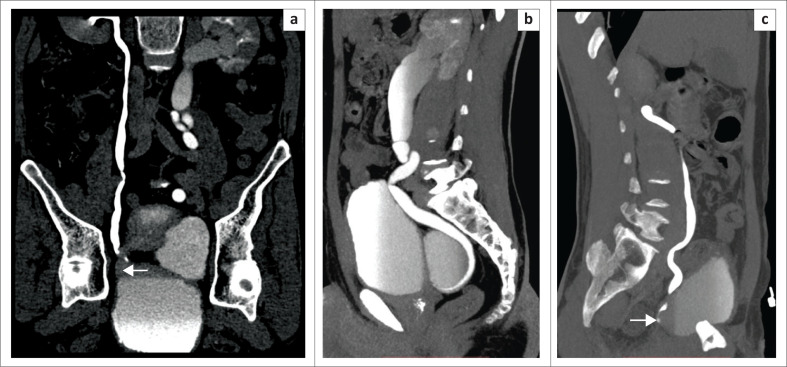
Prone coronal multiplanar reformatted image (a) and sagittal curved maximum intensity projection reformatted images (b – left and c – right) confirm the findings of bilateral hutch diverticula with the ureters emptying into them. (Right diverticula is not filled with contrast and is indicated by the arrow). There is gross hydro-uretero-nephrosis on the left side (secondary to vesicoureteric reflux).

In view of the setting of blunt trauma and continuing pain in abdomen, contrast enhanced computed tomography (CECT) (Figures 4 and 5) was requested by the clinician. After obtaining a written informed consent, a non-contrast Computed tomography (CT) followed by contrast enhanced CT scan of abdomen was performed using a 64 slice multi-detector computed tomopgraphy (MDCT) scanner – Siemens Definition AS, with non-ionic iodinated contrast medium (Omnipaque 350 mg/mL) administered at a rate of 3 mL/s, acquiring portal venous phase and delayed scans. There were no significant findings suggestive of injury detected in the abdomen. Both kidneys showed symmetrical uptake with normal corticomedullary enhancement and excretion of contrast. The 30 min delayed scan also allowed for assessment of the diverticula, ureters and the VUJs. Well-defined contrast-filled outpouchings were seen at the level of the bilateral VUJs. There was moderate left-sided hydronephrosis with a dilated and tortuous left ureter opening into the diverticulum on the left side. The right kidney and ureter were normal with its distal end inserting into the diverticulum on the right. The wall of the diverticulum was of uniform thickness. The UB bladder did not reveal any wall irregularity, trabeculations or thickening. No debris or calculi were seen. The urine examination was also normal.

The patient was discharged with the advice of regular follow-up in the surgical outpatient department (OPD) and to report earlier if any symptoms develop.

## Discussion

Bladder diverticula are rare clinical entities in both the paediatric and adult populations. They are classified as congenital or acquired. The basic differences between the two are highlighted in Table 1. The basic pathology in both types is herniation of the bladder mucosa through the muscular fibers of the wall,^1^ resulting in a thin-walled outpouching arising from the bladder lumen. Histological examination of surgical specimens after resection show similar findings and further support the theory of ‘hypoplasia of the muscularis layer’ as a cause for the herniation. The wall of the diverticulum is thinned out and empties poorly during micturition due to lack of the muscular layer.^1^ This leads to increased post-void residue with paradoxical enlargement of the bladder diverticulum during micturition. Congenital or primary bladder diverticula usually present during childhood^4^ and occur in the absence of bladder outlet obstruction as opposed to the acquired form. They are associated with congenital syndromes, namely Ehlers–Danlos (type 9) syndrome, Menkeskinky hair syndrome, Cutis Laxa syndrome (Sotos) and Williams–Beuren syndrome.^3,6^ In our patient a focused physical examination was performed to rule out these congenital syndromes.

**TABLE 1:** Differences between congenital (hutch) diverticulum and acquired diverticulum.

Variables	Hutch-congenital diverticulum	Other acquired diverticulum
Age	Paediatric	Elderly
Anatomy	Mucosal herniation	Mucosal herniation
Physiology	Weakening of Waldeyer sheath, Progressive increase in size	Increased pressure in the bladder secondary to distal obstruction
Number; location	Usually postero-lateral at the base of trigone; can incorporate VUJ leading to VUR	Multiple different size diverticula along any wall
Clinical	Asymptomatic or symptomatic	Symptomatic
Imaging	Developmental anomaly; chronic obstruction	Distal obstruction; neurogenic bladder.
Cystoscopy	Smooth bladder wall with diverticula	Trabeculations are seen.
Management	If small or asymptomatic, left untreated. Surgical treatment for complications	Distal obstruction treated, may resolve. Surgical approach if complications develop.
Risk of malignancy	No risk	Risk present
Syndromic association	Present	Absent

VUJ, vesicoureteric junction; VUR, vesicoureteric reflux.

On cystoscopy, congenital diverticula reveal a smooth wall,^7^ whereas the acquired diverticula are associated with multiple bladder trabeculations. The congenital diverticula are usually unilateral but can be bilateral and are usually seen at the base of the trigone, in the vicinity of VUJ (6). On the contrary, the acquired diverticula can arise from any portion of the bladder wall and are usually multiple in number. The congenital variety does not have any risk for malignancy as opposed to the secondary acquired type.^8^

Diverticula located superolateral to the ureteral orifice, without involving the trigone, are referred to as ‘Hutch diverticula’ and are usually associated with vesicoureteral reflux. It is a rare developmental anomaly that develops because of failure of muscle development at or near the ureteral orifice. Initially, a small diverticulum is formed because of herniation of bladder mucosa through the weakest point of the bladder musculature,^6^ where Waldeyers’ sheath anatomically covers the space between the intravesical ureter and muscular layer of bladder.^9^ The defect eventually enlarges with voiding and finally the ureteral orifices are incorporated into the diverticulum.^10^

There are very few reported cases, mostly in children. The presence of Hutch diverticulum in adults is very rare.^2,11^ Patients with Hutch diverticulum can be asymptomatic or may have diverse symptoms, secondary to urinary stasis. The most common presentation is with urinary tract infections (UTI). Other presentations may include acute urinary retention, bladder stones, enuresis and possible bladder obstruction if the diverticulum enlarges and obstructs the bladder neck distally.^1,7^

Since there is no risk of malignancy associated with the congenital form, if the patient is asymptomatic, no active intervention is required and advice is given regarding frequent micturation and complete evacuation. If the patient presents with recurrent symptoms, surgical management is warranted. The surgical options include open diverticulectomy (intra- or extra-vesical) which may be beneficial in cases of concomitant prostatic enlargement, allowing simultaneous treatment of both entities. The extravesical approach is reserved for patients with large diverticula associated with peridiverticular adhesions or inflammation.^8^ Laparoscopic approach may also be performed as it has the advantage of minimally invasive surgery. Endoscopic transurethral incision of diverticular neck is the treatment option offered to non-surgical candidates.^12^ In patients with VUR, congenital defects are treated with ureteral reimplantation surgery. However, as the acquired form is mostly secondary to an inappropriate increase in detrusor muscle pressure, reimplantation is never performed in these patients prior to managing the detrusor muscle abnormality. Medical management with drugs to avoid recurrent UTI and anticholinergics to maintain the bladder wall overactivity are available.

## Conclusion

Congenital bladder diverticula are very rare in adults and the radiologist should be able to differentiate them from the commoner acquired conditions. If asymptomatic and uncomplicated, they are usually managed conservatively, but in cases with recurrent symptoms or complications, appropriate surgical intervention should be considered.
